# Metabolic control from the endolysosome: lysosome-resident amino acid transporters open novel therapeutic possibilities

**DOI:** 10.3389/fimmu.2023.1243104

**Published:** 2023-09-15

**Authors:** Toshihiko Kobayashi, Noriko Toyama-Sorimachi

**Affiliations:** Division of Human Immunology, International Research and Development Center for Vaccines, The Institute of Medical Science, The University of Tokyo (IMSUT), Tokyo, Japan

**Keywords:** amino acid transporter, solute carrier family 15, endolysosome, mTORC1, inflammation, metabolism, immune cells, therapeutic target

## Abstract

Amino acid transporters are generally recognized as machinery that transport amino acids from the extracellular environment into the cytoplasm. Although their primary function is the uptake of amino acids to supply the cell with nutrients and energy, endolysosome-resident amino acid (EL-aa) transporters possess several unique functions in accordance with their localization in intracellular vesicular membranes. They play pivotal roles in the maintenance of metabolic homeostasis *via* direct involvement in the amino acid sensing pathway, which regulates the activity of mechanistic target of rapamycin complex 1 (mTORC1), a master regulator of cellular metabolism. Additionally, some EL-aa transporters contribute to the maintenance of dynamic homeostasis of endolysosomes, including the regulation of endolysosomal acidity, by carrying amino acids out of endolysosomes. In addition, EL-aa transporters act as a scaffold to gather signaling molecules and multiple enzymes to control cellular metabolism on the endolysosomal membrane. Among EL-aa transporters, solute carrier family 15 member 4 (SLC15A4) is preferentially expressed in immune cells, including macrophages, dendritic cells, and B cells, and plays a key role in the integration of metabolic and inflammatory signals. In this review, we summarize our recent findings on EL-aa transporter contributions to inflammatory and metabolic signaling in the endolysosomes of immune cells by focusing on the SLC15 family, including SLC15A4 and SLC15A3, and discuss their uniqueness and universality. We also discuss the potential of targeting these EL-aa transporters in immune cells for the development of novel therapeutic strategies for inflammatory diseases. Because these transporters are highly expressed in immune cells and significantly alter the functions of immune cells, targeting them would provide a great advantage in ensuring a wide safety margin.

## 1 Introduction

Macrophages (Mϕs) and dendritic cells (DCs) are the most potent phagocytic cells and play a pivotal role in inflammatory responses. These cells initiate inflammatory responses by ingesting potentially dangerous materials such as pathogens and dead cell-derived nucleic acids *via* receptor-mediated endocytosis or pinocytosis/macropinocytosis, followed by processing and sensing of these materials to elicit adequate inflammatory responses (1). A wide variety of inflammatory materials, including pathogen-associated molecular patterns, are recognized by innate immune sensors within the endosome/lysosomal systems (2, 3). Various pathogen sensors, including TLRs and nucleotide-binding oligomerization domain (NOD) proteins 1/2, localize on the surface of endolysosomes, where they mediate inflammatory signals (2). Receptors for cytokines and chemokines also use the surface of endolysosomal compartments for transmitting signals following the capture of their ligands and subsequent receptor endocytosis (4–6). In such cases, the endolysosomal membrane gathers various molecules including adaptors and enzymes (7).

Signaling pathways mediated by molecules recruited to the endolysosomal membrane are mutually regulated and intricately influenced by each other. For example, an inflammatory signal reflects the metabolic/energy condition *via* the mechanistic target of rapamycin complex 1 (mTORC1) and AMP-activated protein kinase (AMPK) activities, which largely modify the expression of gene targets, including cytokines, chemokines, and transcription factors (8, 9). Because of the integration of multiple cues into endolysosomes, cellular responses to inflammation are optimized for infection or tissue damage. Therefore, endolysosomes provide an essential platform for the integration of metabolic and inflammatory signals and are frequently described as signaling hubs (7, 9). As this concept gains traction, endolysosome-resident amino acid (EL-aa) transporters, which play central roles in mediating both amino acid flow and scaffolding of various signaling molecules at the endolysosome, are attracting increasing attention.

**Figure 1:**
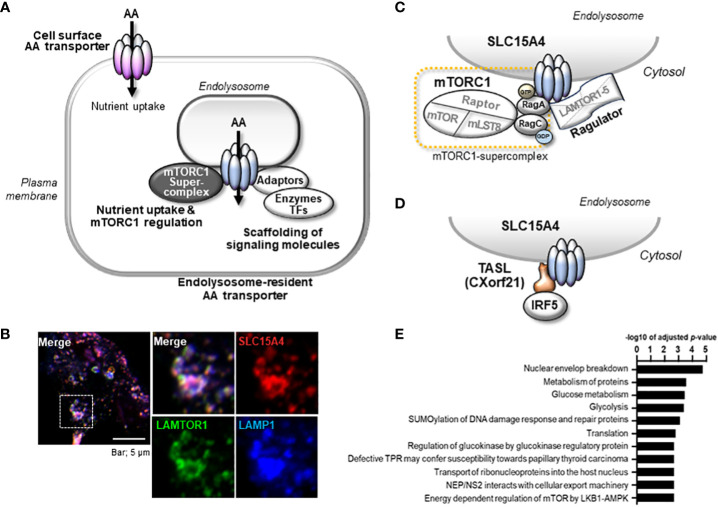
Properties of endolysosome-resident amino acid transporter, SLC15A4. **(A)** Amino acid transporters serve as scaffold proteins in addition to nutrient absorption. In particular, aa transporters localized to the endolysosomal membrane function as scaffold proteins for nutrient/metabolic regulators such as mTORC1 and inflammatory signal-mediating molecules. **(B)** SLC15A4 localizes to the endolysosomal compartment with LAMP1 and LAMTOR (partially modified from reference 21) **(C)** SLC15A4 associates with Ragulator and mTORC1 supercomplex (Complex consisting of mTORC1 and Rag proteins). **(D)** SLC15A4 serves as a scaffold for the TASL (Cxorf21) adaptor protein on endolysosomes. **(E)** Glycolytic-related molecules in close proximity to SLC15A4 have been identified by BioID (11).

The major significance of amino acid transporters is their uptake of amino acids as nutrients and energy sources for cells, and an incredible number of amino acid transporters have been identified (10). Among them, EL-aa transporters possess unique characteristics attributable to their localization in intracellular vesicular compartments (**Figure 1A**). By transporting amino acids from intracellular vesicular compartments to the cytosol or acting as an amino acid sensor, they play pivotal roles in the regulation of cellular metabolism through the activation of mTORC1 (12). Another crucial function of EL-aa transporters is the scaffolding of various signaling molecules, including adaptors, enzymes, and transcription factors (**Figure 1A**). The scaffolding functions of EL-aa transporters are constitutive building blocks for the construction of an endolysosomal signaling platform.

To better understand EL-aa transporters, it is important to acknowledge the pivotal role of the endolysosome system as a signaling platform for controlling cellular metabolic adaptation and energy homeostasis (13). mTORC1 and AMP-activated protein kinase (AMPK) are the master regulators of nutrients and metabolism, respectively, and perform various cellular functions in a manner that is strictly dependent on endolysosomes (7, 14). mTORC1 is activated by amino acids, recruited to the lysosomal membrane, and augments anabolic responses such as protein translation, lysosome biosynthesis, and synthesis of lipids and nucleic acids (15). In contrast, AMPK senses a decrease in intracellular ATP levels and augments catabolic response. AMPK activation suppresses energy consumption by inhibiting mTORC1 activity and promoting ATP production in the mitochondria (14). This mutual control of mTORC1 and AMPK is critical for maintaining the balance between anabolic and catabolic reactions and occurs at the endolysosomal surface (16). The activities of mTORC1 and AMPK strongly affect various cellular functions, including not only cell growth/survival but also inflammatory responses. For example, inhibition of mTORC1 activity severely reduces the production of Toll-like receptor (TLR)9-mediated type I interferon (IFN-I) (17), and AMPK mediates the production of anti-inflammatory cytokines (18).

We are interested in the pathophysiological mechanism of immune cell-specific regulation of the endolysosomal system and have identified an EL-aa transporter, solute carrier family 15 member 4 (SLC15A4), as one of the key players (**Figure 1**) (11, 19–22). SLC15A4 is highly expressed in immune cells, including innate immune cells, and mediates a broad array of signaling functions in endolysosomes. This transporter is a promising therapeutic target in multiple inflammatory diseases, such as lupus and colitis (19, 20, 23, 24), because of its major contribution to both metabolic and inflammatory signaling events (22). In this review, we summarize and discuss the unique functional roles of SLC15A4 and its homolog SLC15A3, which primarily resides in the endolysosomal membrane of immune cells, with a focus on the regulation of inflammatory and metabolic signaling events that occur there.

## 2 Control of mTORC1 by SLC15A4

mTORC1 is a master regulator of nutrition-related signals (25, 26). At the endolysosomal membrane, mTORC1 is activated by the presence of amino acids and requires Ragulator, a membrane-bound regulator complex composed of LAMTOR1–5, Rag GTPases, including RagA–D, and an ATP-dependent vacuolar type (v)-ATPase proton pump (8). Amino acid sensing is mediated by cytosolic sensors, such as leucyl-tRNA synthase (LRS) (27), sestrins (28), and CASTOR1 (29), or by EL-aa transporters, such as SLC36A1 (PAT1) and SLC38A9 (20, 30–32), leading to the activation of Rag GTPases. The active forms of Rag GTPases further recruit mTORC1 to the endolysosome membrane, allowing mTORC1 to exert its function. Since the molecular mechanism of mTORC1 activation by cytosolic amino acid sensors has been well summarized elsewhere (26, 33), we focused on the role of SLC15A4 in the regulation of mTORC1 activity.

SLC15A4 is a member of the SLC15 oligopeptide transporter family (**Table 1**) and possesses 12-transmembrane spanning regions. SLC15A4 localizes in endolysosomal membranes, where it carries amino acids/oligopeptides from the inside of these compartments to the cytoplasm using a proton gradient as a driving force (34). More specifically, SLC15A4 is localized in the LAMP1-positive compartments (**Figure 1B**) (19), which include late endosomes and lysosomes. However, in many previous studies using immune cells, lysosome-specific markers, such as β-galactosidase, were not used to precisely discriminate between late endosomes and lysosomes. Therefore, for convenience, we have used the term “endolysosomes” to indicate LAMP1-positive compartments.

**Table 1:** Characteristics of the SLC15 family transporters.

Gene name (Protein name)	Aliases	Substrate(s) identified	Tissue/Cellular expression substrate(s)	Subcellular localization	Reported function(s)	Implication in human disease(s)
SLC15A1 (PEPT1)	Oligopeptide transporter 1. H+-peptide transporter 1	Di- and Tri-peptides, protons, beta-lactam antibiotics	Epithelial cells of small intestine, and kidney, pancreas, bile duct and liver	Cell surface (Apical surface)	• Peptide uptake of intestinal epithelial cells	• In flammatory bowel disease S117N SNP)
SLC15A2 (PEPT2)	Oligopeptide transporter 2, H+-peptide transporter 2	Di- and Tri-peptides, protons, beta-lactam antibiotics	Apical surface of epithelial cells of kidney and choroid plexus; neurons, astrocytes (neonates), lung, mammary gland, spleen, enteric nervous system, macrophages	Cell surface (Apical surface)	• Nutritional reabsorption in kidney • Transport of neuropeptides in choroid plexus • Transport of bacterial peptide in macrophages	• Porphylia-associated kidney disease
SLC15A3 (PHT2)	Peptide/histidine transporter 2 (PHT2), PTR3	Di- and tri- peptides, Tri-DAP, MDP protons, histidine	Lung, spleen, thymus, intestine (weakly in brain, liver, adrenal gland, heart) macrophages	Endolysosome (LAMP 1-positive compartment)	• L-H is transport • MDP, Tri-DAP transport • Proinflammatory cytokine production • Antiviral response • NOD2-mediated cell death	• Cancer • Inflammatory bowel disease
SLC15A4 (PHT1)	Peptide/histidine transporter 1 (PHT1), PTR4	Di- and tri-peptides, Carnosine, Tri-DAP. MDP protons, histidine	Brain, eye, intestine (weakly in lung and spleen) dendritic cells, B cells, macrophages, mast cells	Endolysosome (LAMP1-positive compartment)	• L-His transport • mTORC1 activation • Regulation of type I interferon production • Metabolic regulation • Transport of bacterial peptide in macrophages	• SLE • Inflammatory bowel disease • Psoriatic inflammation
SLC15A5		Unknown	Fat, brain, testis	Unknown		

Unlike other ubiquitous EL-aa transporters, such as SLC36A1 and SLC38A9, SLC15A4 is highly expressed in immune cells, including Mϕs, B cells, and DCs, particularly plasmacytoid DCs (19, 20, 35). This transporter preferentially carries histidine and histidine-containing di- and oligo-peptides (19, 36), leading to the speculation that this transporter has functions other than nutritional absorption. It has been reported that SLC15A4 transports carnosine (N-β-Alanyl-L-histidine) (36), L-Ala-γ-D-Glu-mDAP (Tri-DAP) (19) and N-Acetylmuramyl-L-alanyl-D-isoglutamine (MDP; muramyl dipeptide) (37, 38), the latter two of which are components derived from bacterial cell walls (**Table 1**). Valacyclovir, a drug used to treat herpes virus infections, is also reported to be transported by SLC15A4 (38). However, the measurement of transporter activity and identification of substrates for EL-aa require caution because of the nature of its localization to intracellular vesicles. In the case of an analysis using transporter-expressing cells, it is necessary to use not only the presence or absence of transporter protein expression but also transporter activity-deficient mutants, since the transfer of substrates into the cell is also affected by transporter-independent routes, such as endocytosis and pinocytosis. In addition to the aforementioned substrates, SLC15A4 can transport several histidine-containing dipeptides (36).

**Figure 2:**
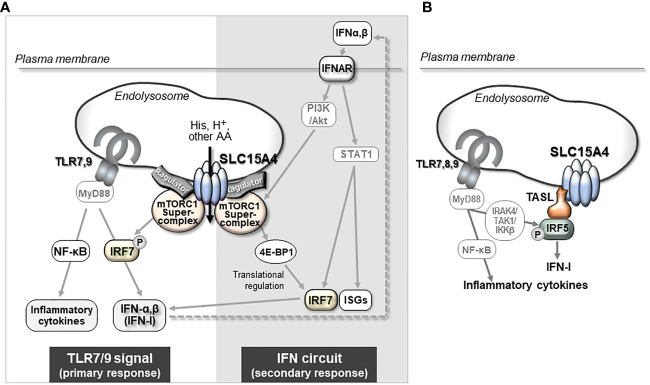
The mechanism by which SLC15A4 regulates TLR7/9-triggered IFN-I production. **(A)** mTORC1-dependent mechanism of IFN-I production by SLC15A4. SLC15A4 regulates the TLR7/9-mediated response by controlling the pH of the endolysosome and controlling mTORC1 activity upon TLR stimulation. The pH of the endolysosome affects the ligand recognition of TLRs, which is essential to initiate the primary signal, and mTORC1 activity is required in the primary response for the activation of IRF7 to induce IFN-I, as well as in the secondary response for IFN-I signaling to amplify IFN-I production. **(B)** The regulatory mechanism of IFN production is dependent on TASL recruitment by SLC15A4. An adaptor protein, TASL, which is recruited to endolysosomes by its association with SLC15A4, mediates the phosphorylation of IRF5 to induce the production of IFN-I.

*SLC15A4* is one of a number of genes associated with diseases such as systemic lupus erythematosus (SLE) and diabetes (39–44), but its physiological roles, apart from the histidine transporter function, have not been examined for many years. Most were disease-associated SNPs, and no genetic defects or mutations in SLC15A4 have been reported to date. It is not clear how these SNPs are associated with disease development, but some SNPs may affect it expression levels (Kobayashi, T. et al. unpublished data). Actually, the analysis using the expression quantitative trait loci (eQTL) database, ImmuNexUT, has shown that the expression of SLC15A4 is increased in individuals with SLE-risk genotypes (45). A further analysis of the functional significance of SNPs in pathogenesis is needed, as with other disease-related SNPs; however, the above observation obtained by an eQTL analysis is consistent with the results of animal model experiments, indicating that SLC15A4 plays a role in lupus pathogenesis. A series of studies using ethylnitrosourea-induced mutant mice (mice with *feeble* mutations) and *Slc15a4* KO mice revealed that this transporter plays critical roles in TLR7- and TLR9-induced IFN-I production and pathogenic antibody production, which contributes to the antiviral response and pathogenesis of lupus (20, 24, 35, 46, 47) (**Figure 2**). TLR7 and TLR9 recognize single-stranded RNA and double-stranded DNA, respectively, at the endolysosomes and activate transcription factors, including nuclear factor (NF)-κB and IFN regulatory factor (IRF)7, to induce the production of inflammatory cytokines and IFN-I (2, 3) (**Figure 2**). These TLR-mediated signaling events are strongly dependent on mTORC1 and endolysosomal pH, because ligand recognition by TLR7/9 requires cathepsin activity (48). In the presence of TLR agonists, SLC15A4 loss causes dysregulation of both endolysosomal pH and mTORC1 activation (20). These disorders result in strong suppression of not only the primary response of TLR7/9 but also the positive feedback regulation of IFN-I (“IFN circuit”) (20). DCs, particularly plasmacytoid DCs, produce large amounts of IFN-I that they receive *via* the IFN-I receptor in an autocrine manner, and are used to transcriptionally activate IFN-stimulated genes (ISGs) (49–51). This IFN-I response is also dependent on endolysosomes; that is, the mTORC1-dependent regulation of the protein translation of IRF7, an IFN-stimulated gene that plays a critical role as a transcription factor of IFN-I production (**Figure 2**). Therefore, SLC15A4 loss strongly suppresses the IFN circuit by reducing mTORC1 activity (20) (**Figure 2**).

Although the precise mechanism by which SLC15A4 regulates mTORC1 activity requires further elucidation, two non-mutually exclusive mechanisms have been identified. One involves dysregulation of the endolysosomal pH, leading to a decrease in mTORC1 activity. More specifically, SLC15A4 loss causes endolysosomal accumulation of histidine (20), which possesses buffering potential through the acid-base catalyzing function of its imidazole ring (52). Because vesicular acidity is required for the v-ATPase function, which is critical for mTORC1 activity, one of the roles of SLC15A4 in mTORC1 activation is to maximize the endolysosomal pH. The second mechanism involves SLC15A4-mediated stabilization of mTORC1 at the endolysosomal membrane. SLC15A4 associates with Ragulator components, including LAMTOR1 and LAMTOR2, and is suggested to be a component of the mTORC1 supercomplex in the endolysosomal membrane (11) (**Figure 1C**). Consistent with this, SLC15A4-deficient cells show an abnormal distribution of Raptor (an mTORC1 constituent molecule) to endolysosomes (20). Therefore, SLC15A4 loss destabilizes the formation of the Ragulator-mTORC1 complex, resulting in a decrease in mTORC1 activity.

## 3 A critical role of SLC15A4 in metabolic reprogramming of Mϕs

Among the EL-aa transporters, SLC15A4 may be unique in its ability to integrate inflammatory and metabolic regulation. In this section, we focus on the function of this transporter in the metabolic reprogramming of Mϕs.

### 3.1 Metabolic reprogramming of Mϕs in the inflammatory response

**Figure 3:**
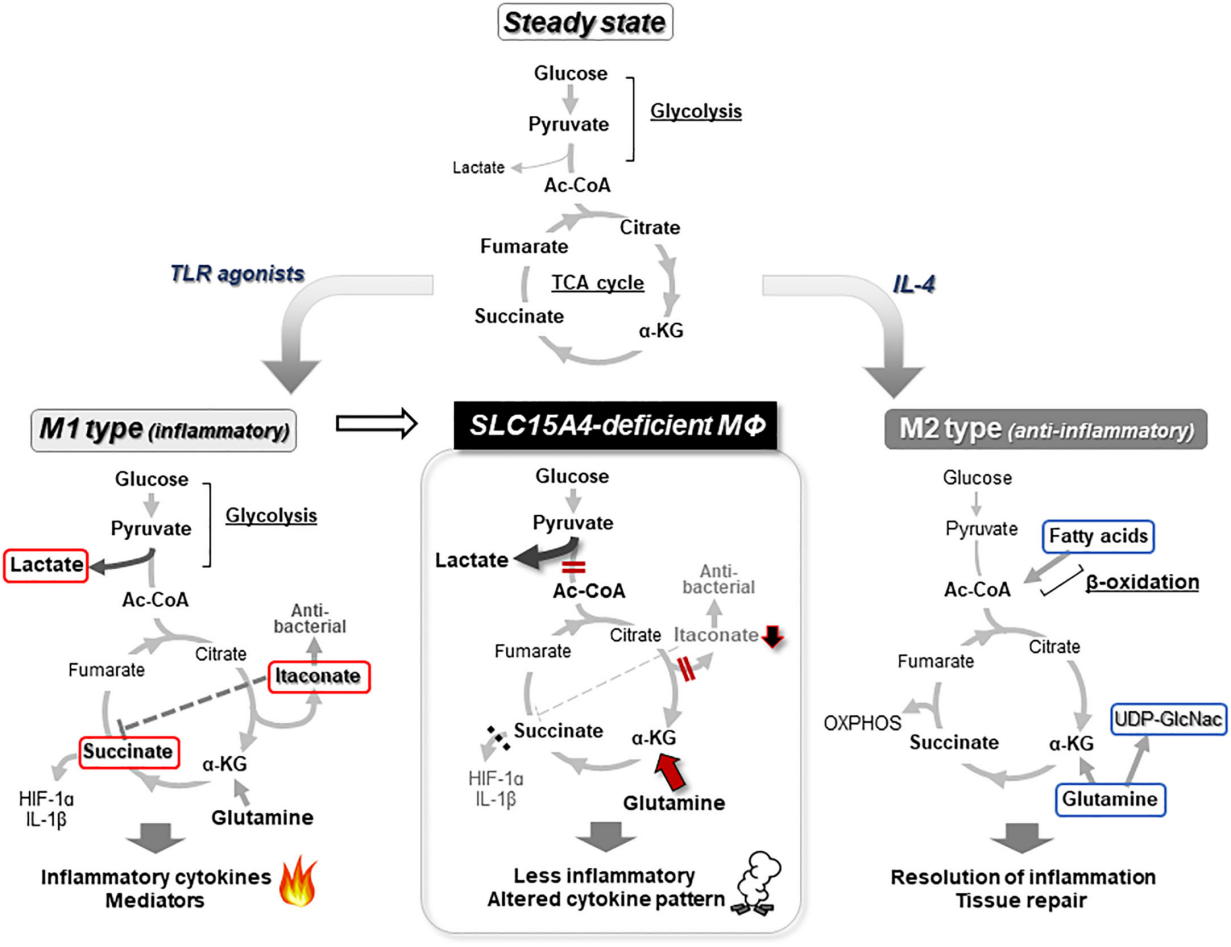
Metabolic reprogramming of macrophages. In a steady state, glucose in Mϕs is metabolized to acetyl-CoA (Ac-CoA) by the glycolysis pathway, and further metabolized for energy production in the TCA cycle (top). When Mϕs receive an inflammatory stimulus, such as that provided by TLR agonists, they shift to an inflammatory M1 type of metabolism in which lactate production is dominant in glycolysis and itaconate is synthesized from aconitate. Because this itaconate inhibits the succinate dehydrogenase that converts succinate to fumarate, the TCA cycle flux is disrupted. The inhibition of succinate dehydrogenase leads to accumulation of succinate, which in turn stabilizes HIF1α, a critical transcription factor for the induction of proinflammatory cytokines, including IL-1β (left). In M2 or anti-inflammatory type Mϕs, metabolism is highly dependent on ATP production by oxidative phosphorylation (OXPHOS) based on fatty acid oxidation (right). In the absence of *Slc15a4*, PDH activity is reduced, thereby impairing the coupling between glycolysis and the TCA cycle, and reducing the supply of Ac-CoA to the TCA cycle (meanwhile, glycolysis is skewed to produce more lactate). The reduced Ac-CoA level in the TCA cycle decreases downstream products, including itaconate and succinate, while the compensatory use of glutamine is increased. This imbalance in metabolism alters the pattern of inflammatory cytokine production (middle) (22). Metabolites that characterize M1 Mϕs are indicated by red lines and metabolites that characterize M2 Mϕs are indicated by blue lines.

Mϕs are innate immune cells that are widely distributed throughout the body, where they recognize and phagocytose foreign microorganisms such as viruses and bacteria to initiate inflammatory responses (53, 54). They also play an important role in the maintenance of tissue homeostasis by promoting the repair of inflamed tissues (54). To fulfill these diverse functions, Mϕs undergo differentiation into unique subsets in a tissue- or situation-dependent manner. The diversity and plasticity of Mϕs are closely associated with metabolic adaptations that reflect the surrounding environment and pro-/anti-inflammatory stimuli in tissues (54, 55). Emerging metabolic analysis tools have revealed that the intracellular metabolism of classic inflammatory M1-type Mϕs and tissue-repairing M2-type Mϕs show large alterations in comparison to steady-state Mϕs, with characteristic metabolism and gene expression patterns (55, 56). This phenomenon of metabolic change is called “metabolic reprogramming” (57). In the steady state, Mϕs convert the pyruvate produced in glycolysis into acetyl-CoA, which is used in the TCA cycle to produce ATP by oxidative phosphorylation of the mitochondria. When Mϕs are stimulated with TLR agonists, for example, their metabolism shifts to one where the rate of conversion from pyruvate to lactic acid increases, and ATP is mainly produced by a pathway known as aerobic glycolysis (**Figure 3**). Simultaneously, in M1 type-Mϕs, TCA cycle flux is disturbed by inflammatory signal-dependent inhibition of isocitrate dehydrogenase and succinate dehydrogenase, which causes the accumulation of intermediate metabolites such as citrate, aconitate, and succinate. Increased aconitate in M1 Mϕs is metabolized to itaconic acid, which alkylates cysteine residues in various proteins and modifies their functions, resulting in antibacterial and anti-inflammatory effects (58). Excess succinate leads to the stabilization of hypoxia inducible factor 1α (HIF1α), which in turn, activates the transcription of glycolytic enzymes and inflammatory cytokines such as IL-1β, resulting in the promotion of the inflammatory response (55, 57) (**Figure 3**). Conversely, M2-type Mϕs, which are believed to be responsible for anti-inflammatory (pro-resolving) functions, are highly dependent on oxidative phosphorylation of mitochondria through fatty acid oxidation to produce ATP (55, 59) (**Figure 3**). In addition, M2 Mϕs showed increased glutamine use in the TCA cycle in comparison to M1 Mϕs, as well as the increased synthesis of glutamine-related metabolites and UDP-GlcNAC (59).

### 3.2 Critical role of SLC15A4 in glycolysis-TCA coupling

**Figure 4:**
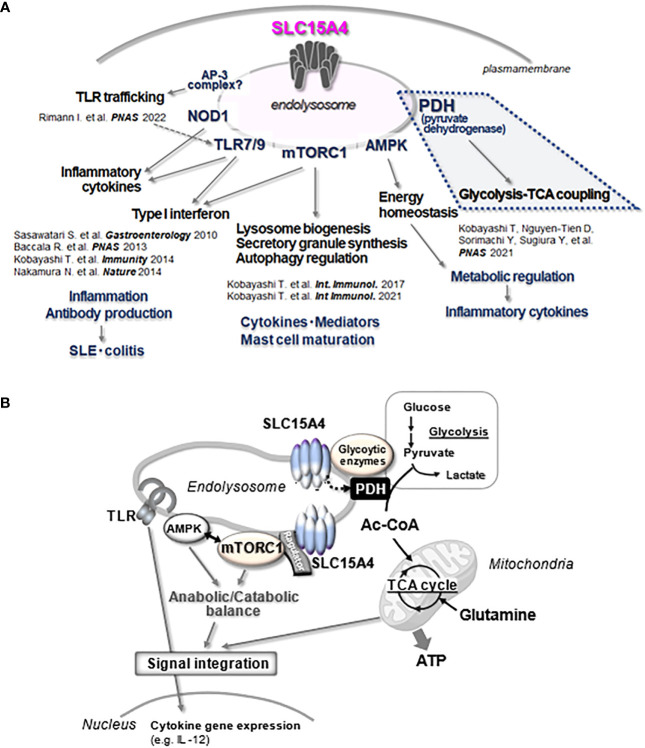
Multitasking of SLC15A4 at the endolysosome. **(A)** The various functions of SLC15A4 in endolysosomes reported in the present study are illustrated. **(B)** The part of the diagram shown in A, especially the part related to metabolic control (blue dotted line), is illustrated in **(B)** In its scaffolding function, SLC15A4 gathers various types of molecules to form complexes on the endolysosomal membrane, which enables efficient bridging between glycolysis and the TCA cycle for ATP (energy) production, directing metabolic adaptation during the inflammatory response. In its mTORC1 activation function, SLC15A4 closely associates with Ragulator and the mTORC1 supercomplex to regulate its activity, which is important for the integration of TLR inflammatory signals and metabolic signals. The inflammatory response of the induced expression of cytokine genes is one outcome of this integration.

As described above, SLC15A4 is required for mTORC1 activation. Notably, SLC15A4-deficient mice do not show any apparent defects in the proliferation or differentiation of immune cells in the steady state (19, 20), strongly suggesting that SLC15A4-dependent mTORC1 regulation is important under inflammatory conditions but not under steady-state conditions. Although mTORC1 suppresses autophagy (60), SLC15A4 loss does not augment starvation-induced autophagy (11). DCs with SLC15A4 knockdown (KD) can elicit an autophagic response under amino acid-starved conditions, as evidenced by the appearance of equivalent numbers of LC3-containing puncta in comparison to controls. Interestingly, however, the initial numbers of LC3 puncta diminished soon afterward in SLC15A4 KD DCs, indicating that SLC15A4 is necessary to sustain the autophagic response (11). These observations provided important insights into the potential role of this transporter in the regulation of glucose metabolism. In the amino acid-starved conditions that are used to induce autophagy, cells are cultured in a medium such as Earle’s Balanced Salt Solution in which glucose is the only carbon source. Under such conditions, SLC15A4 KD DCs showed a severe decrease in mitochondrial membrane potential, indicating that SLC15A4 is critical for glucose-dependent oxidative phosphorylation (11). In parallel, the contribution of SLC15A4 to glucose metabolism was also suggested by the proximity-dependent biotin identification (BioID) method (22). The BioID method involves fusing the *Escerichia coli*-derived BirA protein (a biotin ligase) with a target molecule and expressing it in cells, thereby promiscuously biotinylating the surroundings of the target molecule. After collecting the biotinylated proteins with streptavidin beads and identifying them by mass spectrometry, candidate molecules that interact with the target molecule can be identified (61). Using this method, glycolytic enzymes and members of the glycolytic pathway, together with mTOR-related molecules, were enriched as molecules that possibly interact with SLC15A4 (**Figure 1D**) (22). Using metabolic approaches, including seahorse flux and fluxome analyses, which use stable isotope-labeled carbon sources, such as ^13^C-glucose and ^13^C-glutamine, we revealed that SLC15A4 is required for the conversion of glucose-derived pyruvate to acetyl-CoA, which is a bottleneck reaction that connects glycolysis and the TCA cycle (22). This reaction is mediated by pyruvate dehydrogenase (PDH) (62), the activity of which is negatively regulated by the phosphorylation of the E1α subunit (PDHA) by PDH kinase (63). SLC15A4-deficient cells showed enhanced PDHA phosphorylation and, consequently, diminished PDH activity (22) (**Figure 4B**). PDH kinase activity is regulated by mitochondrial metabolites, including NADH, ATP, and acetyl-CoA (64), suggesting that the production of these metabolites is also influenced by the presence or absence of SLC15A4. Although the molecular mechanism by which endolysosomal membrane-localized SLC15A4 regulates PDH activity localized within mitochondria has yet to be elucidated, these findings indicate that the SLC15A4-mediated regulation of endolysosomal functions is not limited to endolysosomes and may impact other organelles, including mitochondria.

### 3.3 Metabolic licensing of M1 type-Mϕs by SLC15A4

What is the physiological significance of SLC15A4-mediated regulation in the coupling of glycolysis and the TCA cycle? The flux of glucose-derived carbon into the TCA cycle is largely influenced by inflammatory stimulation in Mϕs (56). As described above, Mϕs change their metabolism to an aerobic glycolysis-dominant state upon receiving an M1-inducing inflammatory stimulus such as lipopolysaccharide. In parallel, Mϕs adapting to an M1-type metabolism also produce the anti-microbial metabolite itaconate from citrate (55, 56) (**Figure 3**). In the absence of SLC15A4, the production of itaconate decreases and the TCA cycle becomes dysregulated due to a decreased supply of acetyl-CoA to the TCA cycle (22). Interestingly, the insufficient supply of glucose-derived acetyl-CoA to the TCA cycle in SLC15A4-deficient Mϕs is compensated for by an increased influx of glutamine (22) (**Figure 3**, middle panel). Thus, SLC15A4 could be considered as a gatekeeper of glutamine flux into the TCA cycle in Mϕs.

Depending on the circumstances, cells preferentially use different types of carbon fuel such as glucose, glutamine, and fatty acids to produce ATP. The choice of fuel has a large impact on gene expression and the consequent cellular functions, probably due in part to the influence of altered metabolites, as observed in the example of HIF1 regulation (65). The altered metabolic availability of glucose and glutamine as a result of SLC15A4 loss also has a large impact on the inflammatory response in Mϕs; that is, they exhibit the severely decreased expression of *Il12b* (also known as *p40*) (22). The IL-12 family are cytokines composed of heterodimers, in which two β chains encoded by *Il12b* and *Ebi3*, are associated with at least four different α chains. IL-12 family members encoded by *Il12b* (IL-12, IL-23) are considered inflammatory, whereas those encoded by *Ebi3* (IL-27, IL-35) are considered regulatory (66, 67). SLC15A4 is selectively required for the expression of *Il12b* but not for the expression of *Ebi3*; thus, SLC15A4 likely plays a role in shifting the IL-12 family to more inflammatory molecular species by balancing the expression of inflammatory *Il12b*/*p40* and regulatory *Ebi3*.

Interestingly, the pharmacological inhibition of glutaminase using compound 968 increased the expression of *Il12b*, whereas mimicking augmented glutaminolysis by adding α-ketoglutarate suppressed the expression of *Il12b*, with the latter being a phenocopy of SLC15A4 deficiency (22). Therefore, the balance between the pro- and anti-inflammatory profiles of the IL-12 family appears to be strictly regulated by the carbon source flowing into the TCA cycle. SLC15A4 limits the flow of glutamine into the TCA cycle by controlling PDH activity, which metabolically licenses M1-type Mϕs to encourage the production of inflammatory IL-12 species (22).

Another important function of SLC15A4 in the metabolic licensing of M1 macrophages is the SLC15A4-dependent resistance to metabolic stress caused by the limited availability of certain nutrients. In SLC15A4-deficient Mϕs, the mitochondrial respiratory function is significantly affected by extracellular nutrient conditions (22). In SLC15A4-deficient Mϕs, impaired AMPK activation is induced by a TLR9 agonist, and thus SLC15A4 is required for energy homeostasis in Mϕs under inflammatory conditions (22). Although the mechanism still needs to be analyzed in detail, SLC15A4 co-precipitates with mTORC1 and AMPK, suggesting that these metabolic regulators probably exist in close proximity to SLC15A4 (22). Inflammatory responses are tightly coupled with the regulation of anabolic and catabolic balance through the regulation of mTORC1 and AMPK (68, 69), and endolysosomes are precisely where crosstalk between inflammatory and metabolic signals occurs. SLC15A4, which localizes to such vesicular spaces and associates with AMPK and mTORC1 to influence their activities, is a key molecule in the integrated regulation of inflammatory and metabolic signals (**Figure 4A**).

## 4 Roles of SLC15A3 in inflammatory responses

SLC15A3, another member of the SLC15 family, is also expressed in endolysosomes, where it is involved in inflammatory signaling (70). Human SLC15A3 is structurally similar to SLC15A4 (homology = 47.4%) (71), and transports histidine and certain oligopeptides from the endolysosomal lumen to the cytosol using a proton gradient (72). Although SLC15A3 is highly expressed in innate immune cells, including neutrophils, DCs, and Mϕs, and is further induced by inflammatory stimuli, such as lipopolysaccharide (73), the expression levels of SLC15A4 are stable and largely unaffected by inflammatory stimuli, such as TLR agonists. Neutrophils possess the high expression of both SLC15A3 and SLC15A4 in the steady state. Other innate immune cells, such as DCs and Mϕs, express different levels of SLC15A3 depending on the organ and inflammatory situation. The analysis of SLC15A3-deficient mice revealed that this transporter is involved in the regulation of inflammatory signals such as NOD2 in endolysosomes (70). NOD2 is a sensor of MDP that mediates the induction of inflammatory responses (74). Upon bacterial infection, engulfed bacteria are killed and digested in endolysosomes, followed by the release of MDP from bacterial particles. SLC15A3 transports MDP from the inside of the endolysosome to the cytosol and simultaneously recruits NOD2 and its effector kinase, receptor-interacting serine/threonine kinase 2 (RIPK2), to the endolysosomal membrane, enabling bacterial sensing and signal transduction by NOD2. As expected, Mϕs derived from SLC15A3-deficient mice show a reduction in the production of inflammatory cytokines (e.g., IL-6 and IL-1β) induced by MDP (70). Additionally, SLC15A3 positively regulates the stimulator of IFN genes (STING)-mediated production of IFN-I through its association with cytosolic RNA helicase DDX41, mitochondrial antiviral-signaling protein (MAVS), and STING (75). The precise mechanism by which SLC15A3 regulates this signaling pathway must be further elucidated to clarify the significance of its transporter and scaffolding functions in inflammatory responses.

## 5 Significance of SLC15A4 scaffolding function in inflammatory response

The scaffolding functions of EL-aa transporters may be more important than previously understood. Our BioID analysis demonstrated that several proteins, including mTORC1-related molecules, are in close proximity to SLC15A4, with some being associated with it (22, 76). It was also reported that SLC15A4 associates with “TLR adaptor interacting with SLC15A4 on the lysosome” (TASL) (**Figures 1D**, **2B**) (77), which is encoded by *CXorf21*, a gene associated with SLE (78). TASL is an innate immune adaptor for TLR7, TLR8, and TLR9 signaling and mediates IFN-I production by recruiting transcription factor IRF5 to endolysosomes, which is necessary to induce its phosphorylation in human Mϕ and dendritic cell lines (**Figure 2B**) (77). Although it remains unclear whether SLC15A4 transporter activity is required for recruiting IRF5, the SLC15A4–TASL interaction illustrates the scaffolding role of this amino acid transporter.

Recently, the structure of SLC15A4 was reported at 3 Å resolution using a synthetic nanobody, but not as an SLC15A4-TASL complex (https://doi.org/10.21203/rs.3.rs-2646698/v1). In the AlphaFold model used to predict the structure of the SLC15A4-TASL complex, it is suggested that the N-terminus of TASL binds to SLC15A4. In addition, a recent study using fusion proteins of TASL with SLC15A4 and other lysosomal-localized proteins demonstrated that the phosphorylation of IRF5 and TLR7-mediated IFNβ production can be induced without SLC15A4 if TASL is localized to the lysosome (79). However, the intact complex of SL15A4 and TASL was not analyzed in either study, probably because of the protein stability of TASL, and further detailed and direct observations are needed to understand the interactions of these proteins under physiological conditions.

Another scaffolding example is the association of transporter SLC15A3 with a wide variety of intracellular pathogen sensors, including NOD2, DDX41, MAVS, and STING, to regulate inflammatory signaling (75), as described above. These findings were obtained in forced expression experiments using 293T cells, and it is unclear whether they are associated under physiological conditions and whether the association is direct or complex formation mediated by other molecules (including membrane lipids). The role of transporters as scaffolding proteins requires further validation, including the collection of structural knowledge of association modes. EL-aa transporters often interact or form complexes with a variety of molecules, as observed in BioID and biochemical analyses of SLC15A4 (22). Although not specific to immune cells, SLC38A9, a well-characterized EL-aa transporter, forms a complex with mTORC1, Ragulator, and v-ATPase and plays an essential role in the activation of mTORC1 by amino acids (80). Although the distinction between the scaffolding function and complex formation may be difficult, interactions between transporters and other molecules on the endolysosomal surface may have important unknown functions. The multifunctionality of EL-aa transporters, transporters, and scaffolding functions may be one way to distinguish them from other amino acid transporters.

## 6 SLC15A4 and SLC15A3 as promising therapeutic targets

SLC15A4 and SLC15A3 are highly promising therapeutic targets for inflammatory diseases because endolysosomal dysfunction disturbs effector functions not only in Mϕs but also in multiple immune cells and sometimes strongly suppresses inflammatory responses. An existing drug, hydroxychloroquine (commercially known as Plaquenil), which efficiently perturbs the lysosomal function, strongly inhibits inflammatory responses, including the production of inflammatory cytokines and IFN-I (81). However, the indiscriminate action of this drug on all cell types frequently causes severe side effects (82, 83). In this context, targeting the SLC15A4 transporter would provide a great advantage because it is highly expressed in immune cells and can preferentially modify immune cell functions, thereby ensuring a wide safety margin. In disease models, the alleviation of SLE through SLC15A4-deficiency is theoretically reasonable because SLC15A4 is primarily important in the production of IFN-I, which is a critical cytokine in the pathogenesis of SLE (20, 23, 24). SLC15A4 deficiency has already been shown to alleviate disease in a murine colitis model, in which loss of SLC15A4 causes the diminished production of inflammatory cytokines; this is mainly induced by TLR9 (19). Additionally, NOD1-mediated IL-1β production in Mϕs was almost completely suppressed by the loss of SLC15A4 (19), which is attributable to the function of SLC15A4 as the primary transporter of the NOD1 ligand Tri-DAP from endolysosomes to the cytosol. These observations lend further support to SLC15A4 as a highly promising therapeutic target for SLE and probably colitis. A strategy for targeting SLC15A4 must consider whether its transporter function or its scaffolding function (or both) is important.

SLC15A3 is also a promising target for inflammatory diseases, because its expression is almost entirely limited to immune cells (73). Given that SLC15A3 regulates the inflammatory signaling of NOD2, MAVS, or STING, which are associated with inflammatory diseases (namely Crohn’s disease (84), SLE (85) and STING-associated vasculopathy with the onset of infancy (86), respectively), targeting SLC15A3 might offer the added benefit of reduced side effects.

Other ubiquitous EL-aa transporters, such as SLC38A9 and SLC36A1, may also be therapeutic targets in oncology because of their important roles in cancer cell proliferation and tumor formation. SLC38A9 is required for the activation of mTORC1 signaling and cell growth in several pancreatic cancer cell lines (87). SLC38A9 also plays a role in inflammatory signaling, and its modulation of mTORC1 influences the cytokine expression profile (88). Given the importance of mTORC1 in shaping the outcomes of inflammatory signaling (68), targeting SLC38A9 or SLC36A1 might also be an effective strategy to control inflammation. However, the safety margins of drugs targeting these transporters can be minimized by their ubiquitous expression (31, 89).

## 7 Discussion

The function of the endolysosome as a signaling hub is crucial for inflammatory responses, particularly in innate immune cells. Endolysosome-dependent regulation includes not only pathogen sensor-mediated signaling but also metabolic regulation, such as stimulation-dependent metabolic adaptation. Once inflammation occurs, the nutritional status and oxygen supply of the environment in which the immune cells are located changes rapidly. Immune cells must adapt to these changes while performing functions optimized for infectious inflammatory stimuli. In this sense, the integrated regulation of infection and metabolic signals in the endolysosome is fundamental to the immune cell function. The EL-aa transporter SLC15A4 (and perhaps SLC15A3), which plays a fundamental role in this process, is an important functional molecule and candidate therapeutic target for controlling infectious inflammatory responses. SLC15A4 plays a pivotal role by transporting amino acids and scaffolding various signaling molecules. It physically associates with the mTORC1 supercomplex and directly contributes to the regulation of mTORC1 activity, through which metabolic and inflammatory signals are integrated, generating optimal outcomes under different conditions of inflammation (**Figure 4**). In cells such as Mϕs, which utilize the endolysosomal system for diverse functions such as bactericidal, antigen processing, and metabolic adaptation, there are likely to be novel mechanisms for the EL-aa transporter-dependent regulation of endolysosomal functions.

Multiple transporters are involved in mTORC1 regulation, some of which are expressed ubiquitously and some of which are preferentially expressed in immune cells. What is the significance of the involvement of these transporters? A partial explanation can be found in the multi-hub model of the preferred amino acid substrates proposed by Goberdhan et al. (12). In this model, each transporter forms a hub comprising the Ragulator/mTORC1 complex in the endolysosome and senses their preferred amino acids to activate mTORC1. Since endolysosomes show heterogeneity in resident molecules, as well as localization and acidity, these transporters may exhibit differential distribution and local sensing of amino acids to precisely regulate mTORC1 activity. mTORC1 activity is regulated by both ubiquitous and immune cell-preferential transporter species. These transporters seem to be used differentially to achieve adequate regulation of mTORC1 functions; that is, ubiquitous transporters are required for fundamental mTORC1 functions, and immune cell-preferential transporters are required for immune cell-specific mTORC1 functions. This notion is supported by the importance of SLC36A1 and SLC38A9 (31, 32, 90) but not SLC15A4 and SLC15A3 in cell proliferation and survival (19, 20). Given the wide range of mTORC1 functions, it is intriguing to consider the possibility that different amino acid transporters preserve mTORC1 activity to bias a particular mTORC1 function. Furthermore, there is cell type-specific regulation of the endolysosome system, particularly in immune cells, in which endolysosomes, known as lysosome-related organelles (LROs), are formed to perform diverse functions such as degradation and secretion (91). In addition, the numbers of lysosomes and LROs in immune cells change in response to inflammatory stimulation. In light of the uniqueness of the endolysosome system in immune cells, it seems feasible that immune cell-preferential EL-aa transporters constitute specialized machinery for mTORC1 regulation.

Previous studies on the EL-aa transporter function have overwhelmingly focused on mTORC1 because of its ability to transport amino acids. However, considering that the activities of mTORC1 and AMPK are closely regulated by each other and that the balance between catabolism and anabolism is dynamically controlled by the nutrient and oxygen environment in which the cells are placed, the function of EL-aa transporters, and the relationship with mTORC1 and AMPK, should be analyzed in greater detail. The association of SLC15A4 with AMPK and the results of BioID of SLC15A4 suggest that the EL-aa transporter may exist as a diverse complex of various functional molecules, possibly depending on endolysosomal trafficking and its location. The knowledge accumulated from EL-aa transporter research may greatly expand the concept of transporters in the future.

EL-aa transporters have been identified and functionally analyzed using various genetic, biochemical, and other methods. Crystallographic analyses and pharmacological approaches using liposomes have improved our understanding of their mechanisms of action and physiological relevance (92, 93). However, aspects of EL-aa transporters, such as their mode of intracellular transport, precise distribution, and substrate selectivity, remain poorly understood. Moreover—with the exception of SLC38A9—the precise mechanisms through which EL-aa transporters are involved in mTORC1 regulation remain unclear, and the physiological significance of the interactions between these lysosomal transporters and various other lysosome-resident proteins is an unexplored field of research. Investigating the functions of EL-aa transporters has proven to be technically difficult; however, the use of a proteomic analysis to identify interactors could reveal unexpected functions of these transporters, as reported above. Another important factor for understanding the mode of action of an EL-aa transporter is the quality of lipids in the endolysosomal membrane. Although sphingomyelin and ceramide are enriched in the endolysosomal membrane (94), the role of these sphingolipids in the function of EL-aa transporters is largely unknown. Combining various experimental approaches, including methods traditionally used for transporter research, structural analysis, and comprehensive omics analyses, will advance our understanding of EL-aa transporters, and might facilitate the design of drugs that harness their functions.

## Author contributions

TK and NT-S collected the information and wrote the manuscript. All authors contributed to the article and approved the submitted version.
